# Persistence of parental‐reported asthma at early ages: A longitudinal twin study

**DOI:** 10.1111/pai.13762

**Published:** 2022-03-12

**Authors:** Elise Margaretha Adriana Slob, Cristina Longo, Susanne J. H. Vijverberg, Toos C. E. M. van Beijsterveldt, Meike Bartels, Jouke Jan Hottenga, Mariëlle W. Pijnenburg, Gerard H. Koppelman, Anke‐Hilse Maitland‐van der Zee, Conor V. Dolan, Dorret I. Boomsma

**Affiliations:** ^1^ Department of Respiratory Medicine Amsterdam University Medical Centres University of Amsterdam Amsterdam The Netherlands; ^2^ Department of Paediatric Pulmonology Amsterdam University Medical Centres University of Amsterdam Amsterdam The Netherlands; ^3^ Department of Clinical Pharmacy Haaglanden Medical Centre The Hague The Netherlands; ^4^ Netherlands Twin Register Department of Biological Psychology Vrije Universiteit Amsterdam Amsterdam The Netherlands; ^5^ Department of Paediatrics Division of Respiratory Medicine and Allergology ErasmusMC – Sophia Children’s Hospital University Medical Centre Rotterdam Rotterdam The Netherlands; ^6^ Department of Paediatric Pulmonology & Paediatric Allergology University Medical Centre Groningen Beatrix Children’s Hospital University of Groningen Groningen The Netherlands; ^7^ Groningen Research Institute for Asthma & COPD (GRIAC) University Medical Centre Groningen University of Groningen Groningen The Netherlands

**Keywords:** asthma, asthma‐like symptoms, children, discordant twin design

## Abstract

**Background:**

Currently, we cannot predict whether a pre‐school child with asthma‐like symptoms will have asthma at school age. Whether genetic information can help in this prediction depends on the role of genetic factors in persistence of pre‐school to school‐age asthma. We examined to what extent genetic and environmental factors contribute to persistence of asthma‐like symptoms at ages 3 to asthma at age 7 using a bivariate genetic model for longitudinal twin data.

**Methods:**

We performed a cohort study in monozygotic and dizygotic twins from the Netherlands Twin Register (NTR, *n* = 21,541 twin pairs). Bivariate genetic models were fitted to longitudinal data on asthma‐like symptoms reported by parents at age 3 and 7 years to estimate the contribution of genetic and environmental factors.

**Results:**

Bivariate genetic modeling showed a correlation on the liability scale between asthma‐like symptoms at age 3 and asthma at age 7 of 0.746 and the contribution of genetics was estimated to be 0.917. The genetic analyses indicated a substantial influence of genetic factors on asthma‐like symptoms at ages 3 and 7 (heritability 80% and 90%, respectively); hence, contribution of environmental factors was low. Persistence was explained by a high (rg = 0.807) genetic correlation.

**Conclusion:**

Parental‐reported asthma‐like symptoms at age 3 and asthma at age 7 are highly heritably. The phenotype of asthma‐like symptoms at age 3 and 7 was highly correlated and mainly due to heritable factors, indicating high persistence of asthma development over ages 3 and 7.

**Abbreviations**
A: additive genetic effects
ALSPAC: Avon longitudinal study of parents and children
APRS: asthma polygenic risk score
C: shared or common environment effects
DZ: dizygotic
E: non‐shared environmental effects
GWAS: genome‐wide association studies
NTR: Netherlands twin register
MZ: monozygotic
SNPs: single nucleotide polymorphisms
PIAMA: prevention and incidence of asthma and mite allergy

**Key Message**

A large part of the variance in liability to parental‐reported asthma‐like symptoms at age 3, and asthma at age 7 was explained by genetic factors.

The high phenotypic correlation between asthma‐like symptoms at age 3 and asthma at age 7, was almost entirely explained by persistence of genetic effects.

## 1 INTRODUCTION

Asthma often originates early in life,^1^, ^2^ and its clinical presentation may be characterized by a remitting or relapsing course.^3^, ^4^, ^5^ Currently, it is difficult to accurately identify asthma onset, because conclusive diagnostic tests suitable for young children are lacking.^2^ Asthma‐like symptoms, that is, wheezing, coughing, and shortness of breath, are common in early childhood, but insufficiently specific to diagnose asthma reliably.^2^ At present, we are unable to predict which infant with asthma‐like symptoms ultimately develops a transient phenotype, for example, remission of asthma‐like symptoms, or persistent asthma, with continuation of asthma‐like complaints up to school age.^6^, ^7^, ^8^, ^9^ Understanding to which extent childhood asthma onset is influenced by genetic and environmental factors and to which extent these contribute to remission and persistence may improve risk‐stratification and facilitate targeted management of children at risk of persistent asthma.

The classical twin study is a common tool to estimate genetic and environmental contributions to complex human traits, including disease, based on the fact that monozygotic (MZ) twins are genetically (nearly) identical and dizygotic (DZ) twins share on average 50% of their segregating genes. Both types of twins share their prenatal environment and grow up in the same family environment. A larger resemblance in MZ compared to DZ twins for a trait or disease is consistent with a genetic contribution to that trait.^10^ A study in Dutch 5‐year‐old twins with asthma‐like symptoms and Swedish 9‐to‐12‐year‐old twins with mid‐childhood asthma^11^, ^12^, ^13^ suggested that genetics accounted for 60%–90% of variance in susceptibility to asthma. However, cross‐sectional twin studies cannot address the childhood‐asthma course and do not reveal contributions of genetics and environment to persistence or remission. High heritability at various ages does not necessarily imply that the same genes are active.^14^

Only a few genetic studies analyzed asthma‐like symptoms in children.^15^, ^16^ In Avon Longitudinal Study of Parents and Children (ALSPAC, *n *= 7,045 children), 244 independent single nucleotide polymorphisms (SNPs) and 13 SNPs from 17q21 were associated with asthma and investigated whether these were associated with six wheezing phenotypes.^15^ SNPs encoding *ORMDL3*, *IKZF3*, and *GSDML* were associated with intermediate‐onset and persistent, but not early‐onset wheezing.^15^ The SNPs from 17q21 accounted for most associations with doctor‐diagnosed asthma.^15^ A meta‐analysis in 2,007 children in Prevention and Incidence of Asthma and Mite Allergy (PIAMA) and 7,247 in ALSPAC found that SNPs encoding *IL33* and *IL1RL1* were associated with asthma, intermediate and late‐onset wheeze phenotypes.^16^

In this large, longitudinal twin study, we aimed to estimate the extent to which genes and environment contribute to asthma‐like symptoms at 3 and 7 years and in persistence of the asthma phenotype from age 3 to 7. Our main interest lies in genetic persistence as expressed in the genetic correlation across these ages and contribution of genetic effects to these phenotypic correlations.

## 2 MATERIALS AND METHODS

### 2.1 Study design and population

We conducted a cohort study of twins aged 3 and 7 years included in the Netherlands Twin Register (NTR), a birth cohort initiated in 1987.^17^ Twins are registered by their parents after birth and recruited with help of a Dutch commercial organization and the Dutch Association for Parents of Multiples. Data are collected by surveys at 0, 2, 3, 5, 7, 9/10, and 12 years. The response rates were 40%–75%. Here, we analyzed data collected between 1989 and 2016. Informed consent was obtained from parents or the guardians. The study was approved by the Central Ethics Committee on Research Involving Human Subjects of the VU University Medical Centre, Amsterdam, an Institutional Review Board certified by the U.S. Office of Human Research Protections (IRB number IRB00002991 under Federal‐wide Assurance‐ FWA00017598; IRB/institute codes, NTR 03‐180). All data were anonymized. Survey data were available for 7,329 MZ and 14,212 DZ pairs (including twins with data only for age 3 or 7; there were 2,646 MZ and 4,857 DZ pairs with longitudinal asthma data). We determined twin zygosity from survey questions^18^ or from DNA or blood polymorphisms (29% of same‐sex twin pairs). The survey included questions on eye, hair, and facial color and shape resemblance, and whether twins were mistaken for each other. NTR validation studies showed that 97.2% of the twins were correctly classified.^17^

### 2.2 Outcomes

A child was considered to have asthma‐like symptoms at age 3 and asthma at age 7 based on the survey question: “Can you indicate for each of the following conditions whether it applies?” followed by a checked box for asthma, chronic bronchitis, or chronic non‐specific respiratory disorders. Since pre‐schoolers cannot reliably perform lung function testing and, thus, cannot have an asthma diagnosis confirmed, we refer asthma‐like symptoms instead of asthma at age 3. Clinicians refer to asthma persistence, whereas in genetic models, the statistical term is stability. Here, we use persistence of asthma‐like symptoms at ages 3 and 7.

### 2.3 Asthma polygenic risk score

In addressing genetic continuity, we included an asthma polygenic risk score (APRS), defined based on SNPs identified in previous genetic association studies. To calculate the APRS, we included 26 top hits associated with asthma‐like symptoms or childhood asthma phenotypes from six genome‐wide association studies (GWAS) investigating SNPs associated with childhood‐onset asthma and two candidate‐gene replication studies investigating whether previously reported SNPs were associated with childhood asthma phenotypes (Table S1). The APRS was calculated by counting risk alleles (0/1/2) per SNP. In our study population, 6,694 children (of whom 3,628 MZ, 1,874 same‐sex DZ and 1,192 opposite‐sex DZ) were genotyped on Affimetrix 6.0, Affimetrix Axiom, or Illumina GSA platforms.^17^

### 2.4 Statistical analysis

**FIGURE 1:**
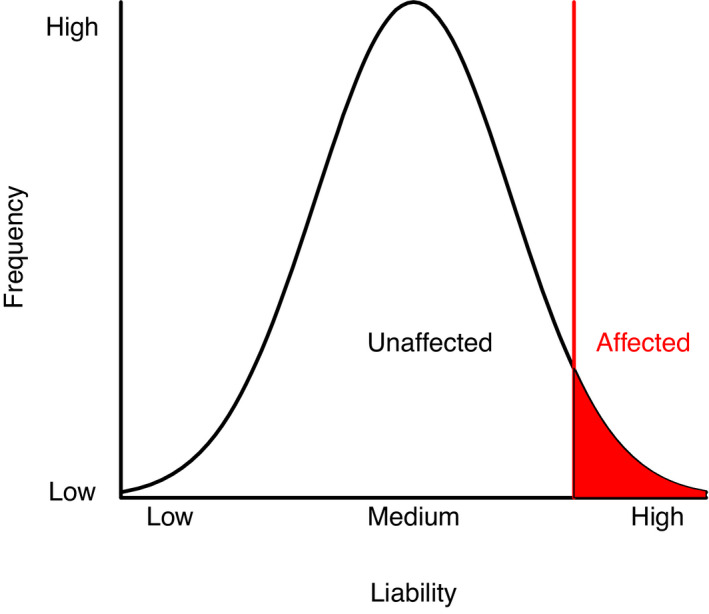
Liability threshold model. A threshold in the liability to disease (x‐axis) determines for an individual their affected/unaffected status of a disease or disorder. A tetrachoric correlation summarizes the resemblance in liability of two individuals such as mono‐ or dizygotic twins

We analyzed longitudinal twin data at ages 3 and 7 by genetic covariance structure analysis^19^ based on a liability threshold model,^20^ where we assume that a continuous liability (or susceptibility) to develop asthma underlies the phenotypic dichotomy (0/1). The liability dimension is assumed to be a normal distribution, which is reasonable as asthma is highly polygenic (i.e., subject to effects of many genes^13^). The threshold value is estimated from the data and depends on phenotype prevalence. Specifically, the estimated threshold equals the standard normal quantile associated with probability of a phenotype value of 1 in the sample. Twin resemblance was summarized by tetrachoric correlations: the correlation between asthma liabilities of twins (Figure 1). MZ and DZ tetrachoric correlations are informative concerning genetic and environmental contributions to individual differences in liability,^13^ as we expect MZ correlations to be higher than DZ correlations when genes contribute to variation in liability.

**FIGURE 2:**
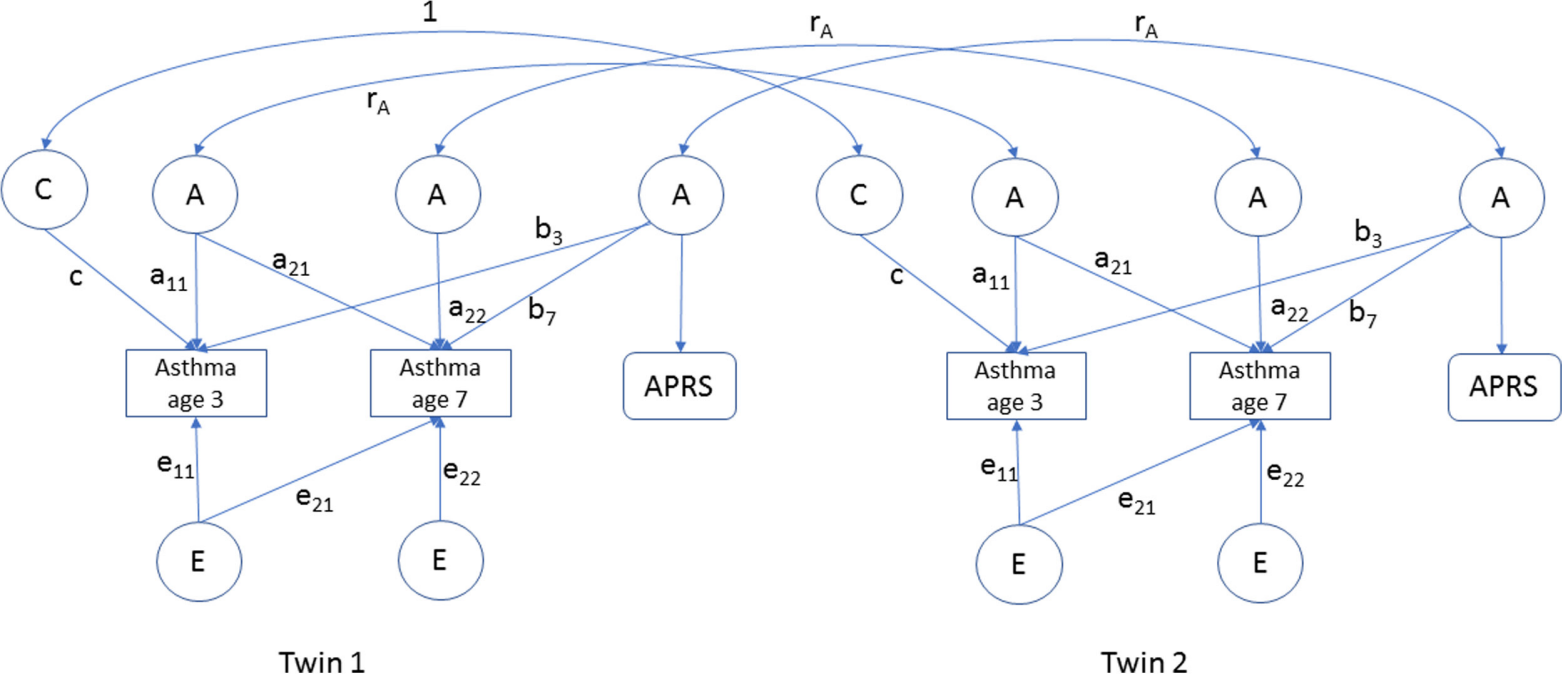
Longitudinal latent factor model including measured asthma outcome data at age 3 and 7 years, and a asthma polygenetic risk score (APRS). A: Additive genetic factor score, C: Shared or common environmental factor score (included at age 3), E: Non‐shared environmental factor score. APRS, asthma polygenetic risk score based on genotype information. r_A_ =the genetic correlation between the factor scores of the two twins and equals 1 for MZ) and 0.5 for DZ pairs

The contribution of additive genetic effects (A), shared or common environment (C), and non‐shared environment (E) to asthma liability variance at age 3 and 7 was estimated by fitting a genetic covariance structure model to the twin data. We obtained estimates of the contributions of A, C, and E to the variance and the covariance of the liabilities at ages 3 and 7. A path diagram is shown in Figure 2, in which A, C, and E contributions are parameterized by means of Cholesky decomposition (a common parameterization of the multivariate ACE twin model). The parameters of the model as included in Figure 2 are associated with the A part: that is, a_11_, a_21_, and a_22_ and likewise for the A and E part. In terms of these parameters, estimates of the total A variance at ages 3 and 7 are a_11_^2^ and a_21_^2^+a_22_^2^, respectively, with similar application to the C and E part, The total liability variance is the sum of these components, that is, a_11_^2^ + c_11_^2^ + e_11_^2^ at age 3, and a_21_^2^+a_22_^2^ + c_21_^2^+c_22_^2^ + e_21_^2^+e_22_^2^, at age 7. Note that the liability is standardized to that the components add up to 1 at age 3 and age 7. This implies that a_11_^2^, a_21_^2^+a_22_^2^ etc. can be interpreted as proportions of liability variance. The contribution of A, C, and E to the phenotypic stability is determined by the products a_21_*a_22,_ c_21_*c_11_, and e_21_*e_11_. In Figure 2, common environment at age 7 was not included in the model because the C variance at age 7 was close to 0. Based on the model, we can obtain the correlation of A at age 3 and A at age 7 as follows: rA = (a_21_*a_11_)/ (a_11_^2^* a_21_^2^+a_22_^2^)^½^. Similarly for E correlation, we have rE = (e_21_*e_11_)/ (e_11_^2^* e_21_^2^+e_22_^2^)^½^.

In addition, Figure 2 included the APRS, which does not vary over time as it depends on a person's genotype, but its contribution at two ages can differ, as indicated by the distinct parameters b_3_ and b_7_. By regressing the asthma liability on the APRS, we estimated contributions of APRS to liability variance at ages 3 and 7, and liability covariance between ages 3 and 7. Parameters b_3_ and b_7_ represent age‐dependent influences of the APRS at ages 3 and 7, respectively. To obtain parameter estimates, we fitted this model using OpenMx in R version 3.5.3 (R Core Team, 2020) by raw‐data maximum likelihood, including all available data. We included gender as a covariate (omitted from Figure 2), to accommodate the main effect of gender on asthma (i.e., gender differences in prevalence of asthma at ages 3 and 7).

## 3 RESULTS

### 3.1 Study population characteristics

For descriptive purposes, we show the different asthma phenotypes in our study population (Table 1). Of the 15,213 individuals with parental report of asthma‐like symptoms at ages 3 and 7, 8 (0.05%) individuals were excluded due to missing zygosity information, 2,154 children were included in one of the three asthma‐phenotype groups: 1) transient asthma (5.6%, i.e., asthma‐like symptoms at age 3, but not at age 7), 2) school‐age onset asthma (3.8%, i.e., no asthma‐like symptoms at age 3, but asthma at age 7), and 3) persistent asthma (4.8%, i.e., asthma‐like symptoms at age 3, asthma at age 7). 13,059 children never experienced asthma (85.8%, i.e., no asthma‐like symptoms at age 3, no asthma at age 7). Within the asthma‐phenotypes there were more males (58.3% (transient asthma), 61.6% (persistent asthma), 56.0% (school‐age onset asthma), compared to never asthma (48.0%)). Children with an asthma phenotype were more likely to have atopy (25.6% (transient asthma), 35.7% (persistent asthma), 30.4% (school‐age onset asthma), versus 13.2% that never had asthma), and were generally less often breastfed compared to never asthma (39.4% (transient asthma), 43.3% (persistent asthma), 44.7% (school‐age onset asthma) versus 36.2% never asthma). In the school‐age asthma onset group more children were without older siblings (89.4%) compared to persistent asthma (47.3%) and transient asthma (41.3%). Characteristics and numbers individuals with asthma‐like symptoms at ages 3 and 7 are provided in Table S2. There were 3,087 children with asthma‐like symptoms at age 3 (1,053 MZ, 1,027 DZ same‐sex and 1,006 DZ opposite‐sex) and 2,273 children with asthma at age 7 (774 MZ, 758 DZ same‐sex and 741 DZ opposite‐sex). The phenotypic correlation in liability between asthma‐like symptoms at ages 3 and 7 was 0.746.

**TABLE 1:** Demographics of individual children in the different asthma phenotypes and ages

	Dynamic phenotypes between age 3 and 7		Stable phenotypes between age 3 and 7	
	Transient asthma (*n* = 847)	School‐age onset asthma (*n* = 573)	Persistent asthma (*n* = 734)	Never asthma (*n* = 13,059)
Zygosity				
MZ	292 (34.5%)	187 (32.6%)	255 (34.7%)	4,630 (35.5%)
DZ same‐sex	277 (32.7%)	305 (53.2%)	252 (34.3%)	4,209 (32.2%)
DZ opposite‐sex	278 (32.8%)	278 (48.5%)	227 (30.9%)	4,214 (32.3%)
Gender				
Male	494 (58.3%)	321 (56.0%)	452 (61.6%)	6,271 (48.0%)
Breastfeeding				
None	334 (39.4%)	256 (44.7%)	318 (43.3%)	4,722 (36.2%)
<2 weeks	96 (11.3%)	44 (7.7%)	80 (10.9%)	1,194 (9.1%)
2–6 weeks	118 (13.9%)	72 (12.6%)	71 (9.7%)	1,925 (14.7%)
6 weeks – 3 months	86 (10.2%)	73 (12.7%)	90 (12.3%)	1,661 (12.7%)
3–6 months	71 (8.4%)	37 (6.5%)	59 (8.0%)	1,243 (9.5%)
>6 months	68 (8.0%)	48 (8.4%)	57 (7.8%)	1,190 (9.1%)
Missing	74 (8.7%)	43 (7.5%)	59 (8.0%)	1,124 (8.6%)
Outside home child care				
None	58 (6.8%)	40 (7.0%)	58 (7.9%)	963 (7.4%)
1–4 h week^−1^	93 (10.9%)	67 (11.7%)	100 (13.6%)	1,947 (14.9%)
5–8 h week^−1^	232 (27.4%)	151 (26.4%)	187 (25.4%)	4,185 (32.0%)
9–16 h week^−1^	172 (20.3%)	110 (19.2%)	142 (19.3%)	3,379 (25.9%)
17–24 h week^−1^	144 (17.0%)	106 (18.5%)	122 (16.6%)	2,808 (21.5%)
>24 h week^−1^	42 (5.0%)	38 (6.6%)	55 (7.4%)	1,094 (8.4%)
Missing	106 (12.5%)	61 (10.6%)	70 (9.5%)	1,890 (14.5%)
Educational attainment mother				
≤9 years	50 (5.9%)	24 (4.2%)	55 (7.5%)	424 (3.2%)
10–12 years	236 (27.9%)	177 (30.9%)	194 (26.4%)	3,215 (24.6%)
<2 years tertiary	368 (43.4%)	233 (40.7%)	324 (44.1%)	5,576 (42.7%)
≥2 years tertiary	193 (22.8%)	139 (24.3%)	160 (21.8%)	3,835 (29.4%)
Missing	0 (0%)	0 (0%)	1 (0.1%)	9 (0.1%)
Educational attainment father				
≤9 years	57 (6.7%)	37 (6.5%)	58 (7.9%)	663 (5.1%)
10–12 years	234 (27.6%)	177 (30.9%)	244 (33.2%)	3,304 (25.3%)
<2 years tertiary	314 (37.1%)	170 (29.7%)	235 (32.0%)	4,573 (35.0%)
≥2 years tertiary	230 (27.2%)	184 (32.1%)	189 (25.7%)	4,445 (34.0%)
Missing	12 (1.4%)	5 (0.9%)	8 (1.1%)	74 (0.6%)
Atopy	217 (25.6%)	174 (30.4%)	262 (35.7%)	1,720 (13.2%)
Missing	184 (21.7%)	118 (20.6%)	157 (21.4%)	2,296 (17.6%)
Older siblings				
None	355 (41.9%)	512 (89.4%)	347 (47.3%)	5,982 (45.8%)
Missing	113 (13.3%)	61 (10.6%)	97 (13.2%)	1,867 (14.3%)

Note

Atopy was based on parental‐reported hay fever or eczema before the age of 5 years old.

### 3.2 Longitudinal genetic analysis

Table 2 includes MZ and DZ tetrachoric correlations for asthma at ages 3 and 7. Based on these correlations, a model including additive genetic and non‐shared environmental effects was selected to estimate heritability and genetic and non‐genetic correlations for asthma at ages 3 and 7, with a contribution of shared environment (C) tested at age 3. The results demonstrate strong genetic contributions to asthma susceptibility: heritability at age 3 was 0.796 and heritability for asthma at age 7 0.904. Shared environment (C) explained 15.7% of the liability variance at age 3. Genetic and environmental correlations for asthma‐like symptoms at age 3 and 7 years in this model were estimated at 0.917 and 0.083, respectively. The results further showed that the APRS did not contribute to the liability variance, in a model with equal regression coefficients at ages 3 and 7, the p‐value was 0.40 (Table S3).

**TABLE 2:** Tetrachoric correlations and estimates for asthma at ages 3 and 7, estimates for the model without asthma polygenic risk score

	MZ (*n*)	MZ cor	DZ same‐sex (*n*)	DZ opposite‐sex (*n*)	DZ cor	Proportion due to A heritability	Proportion due to C	Proportion due to E
						Standardized liability variance components		
Age 3	7,329	0.954	7,177	7,035	0.555	0.796	0.157	0.046
Age 7	7,329	0.904	7,177	7,035	0.452	0.904	0*	0.096
						Liability covariance		
Age 3 and 7	r_liability_: 0.746					R(A) = 0.807	NA	R(E) = 0.934
Contribution to r_liability_						0.685	NA	0.062
Proportions						0.917	NA	0.083

Note

* Fixed to zero. Adjusted for gender.

Abbreviations: AAPRS, asthma risk score; C, shared environment component; cor, correlation; DZ, dizygotic; E, non‐shared environment component; MZ, monozygotic; *n*, number of twin pairs including twins with missing data; NA, not applicable; R(A), genetic correlation; R(E), environmental correlation.

During childhood, asthma prevalence in boys is higher than in girls. Therefore, we investigated whether the heritability for asthma liabilities is sex‐dependent by testing whether the twin correlations differed as a function of sex. Specifically, we carried out a test of rMZFF =rMZMM and rDZMM =rDZM = rDZMF). The last test also includes a test for qualitative sex differences.^21^ Results showed the correlations within MZ and DZ groups did not differ as a function of sex, so we infer that there is no sex moderation at age 3 and age 7 (Supplementary information 2) and also that the same genes are expressed in boys and girls.

## 4 DISCUSSION

Heritability accounted for a large part of the variance in liability to parental‐reported asthma‐like symptoms at ages 3 and 7. The high phenotypic correlation between asthma‐like symptoms at ages 3 and 7 was almost entirely explained by persistence of genetic effects.

The heritability of asthma at 3 and 7 years corresponds with previous twin studies.^11^, ^12^, ^13^ Our finding that environmental factors are not substantially contributing to susceptibility of asthma‐like symptoms at age 3 and 7 aligns with other studies reporting high heritability. A Swedish study in over 25,000 9‐to‐12‐year‐old twins asking whether the child ever had asthma demonstrated that non‐shared environmental contribution was 13% and heritability was 87%. These results were validated with asthma diagnosis data from the National Patient Register.^11^ Thus, our study implies that persistence of asthma symptoms in childhood is more likely due to genetically driven mechanisms. The absence of sex moderation at age 3 and age 7 also corresponds with earlier research showing that there were no sex differences in asthma heritability.^13^

To our knowledge, none of the previous twin studies have incorporated an APRS to their model to estimate contribution of currently known genetic loci to asthma phenotypes and our APRS has not been incorporated in a non‐twin study. We did not find an effect of the APRS which was defined by a count of asthma risk alleles (0/1/2) per earlier identified SNP. This may indicate that childhood asthma is highly polygenic and heterogeneous.^22^, ^23^ We hypothesize that some SNPs may be relevant for one childhood asthma subtype but may not be accounting for another subtype and that a more powerful APRS depends on a next generation of genetic association studies.

A strength of our study is the large cohort including over 20,000 Dutch twin pairs of which over 7,500 twin pairs had longitudinal data describing whether they had asthma‐like symptoms at ages 3 and 7. Our design can estimate the contribution of all genetic and environmental factors (shared and non‐shared). There is uncertainty whether asthma‐like symptoms at young age help predicting childhood asthma onset at school age. Therefore, our main focus was on contribution of genes and environment to asthma persistence over age 3–7 which has yet to be investigated in a bivariate twin model. It was thought that twins are not representative to estimate risk of asthma, but this concern has been addressed previously.^24^ A study in Swedish twins (*n* = 756,363) and singletons (*n* = 456,239) indicated that the observed higher risk of childhood asthma in twins compared to singletons appears to be mediated by differences in gestational age and birthweight between these populations.^24^

This study also had some limitations. Firstly, outcome misclassification may have occurred since clinical data of asthma‐like symptoms and asthma were not available. The asthma‐like symptom phenotype at age 3 is less precise due to difficulty in the diagnosis. Nevertheless, data were retrieved from surveys sent to parents at age 3 and 7, minimizing recall bias. Second, gene‐environment interactions were not investigated in our model. However, in 5‐year‐old Dutch twins overlapping to a large extent with our study population, a dedicated set of analyses tested for interactions with gestational age, time in incubator, breastfeeding, maternal educational attainment, maternal smoking during pregnancy, current parental smoking, presence of older siblings, and of childcare outside home, gene‐environment interactions were barely identified. Several investigated environmental factors did increase risk of asthma, but did not modify genetic influences on asthma, with only one exception, namely prematurity.^25^ These results make it unlikely that gene‐environment interactions lead to overestimation of heritability of asthma development in our model. Third, we assume equal environments for MZ and DZ twins, that is, MZ twins share their early environment in the uterus to the same extent as DZ twins. Violation of this assumption seems unlikely given the fact that this bias has not been observed in other diseases.^26^ Fourth, there may be an overestimation of never asthma, because children with asthma‐like symptoms only at 1–2 years but not at 3 years were assigned as never asthma. Moreover, we could not investigate children under 3 years and environmental factors may play a larger role in this group.

Genes play a substantial role to persistence of asthma over ages 3 and 7, and we could use this knowledge to define high versus low‐risk groups. Prediction of asthma risk and persistence is likely improving with larger GWAS and potentially when results such as (epi)genetic and gene‐gene interaction scores are added to prediction models.

To conclude, our results show that parental‐reported asthma‐like symptoms at age 3 and asthma at age 7 are highly correlated and heritable, indicating high persistence of asthma development over ages 3 and 7. Future studies should elucidate genetics of well‐diagnosed, persistent childhood asthma further and identify mechanisms that could be targets of future interventions.

## CONFLICT OF INTEREST

AHM has received research grants outside the submitted work from Lung Foundation of the Netherlands, GSK, Boehringer Ingelheim and VERTEX, she is the PI of a P4O2 (Precision Medicine for more Oxygen) public private partnership sponsored by Health Holland involving many private partners that contribute in cash and/or in kind (Boehringer Ingelheim, Roche Breathomix, Fluidda, Ortec Logiqcare, Philips, Quantib‐U, Smartfish, SODAQ, Thirona, TopMD and Novartis), and she has served in advisory boards for AstraZeneca, GSK and Boehringer Ingelheim with money paid to her institution. G.H. Koppelman reports grants from Lung Foundation of the Netherlands, TEVA the Netherlands, UBBO EMMIUS Foundation, TETRI Foundation, GSK, and VERTEX, outside the submitted work; and G.H. Koppelman participated in a global advisory board on pediatric asthma for GSK and an advisory board meeting to PURE‐IMS. C. Longo, C.E.M. van Beijsterveldt, S.J.H. Vijverberg, D.I. Boomsma, C. Dolan, M. Bartels, J.J. Hottenga, M.W. Pijnenburg and E.M.A. Slob report no conflict of interest for this study.

## AUTHOR CONTRIBUTIONS

**Elise Margaretha Adriana Slob:** Conceptualization (equal); Formal analysis (supporting); Investigation (lead); Methodology (supporting); Visualization (equal); Writing – original draft (lead); Writing – review & editing (equal). **Cristina Longo:** Conceptualization (lead); Formal analysis (supporting); Investigation (supporting); Methodology (equal); Supervision (lead); Writing – review & editing (equal). **Susanne Vijverberg:** Conceptualization (supporting); Funding acquisition (supporting); Supervision (supporting); Writing – review & editing (supporting). **Toos C.E.M. van Beijsterveldt:** Data curation (equal); Investigation (supporting); Project administration (equal); Supervision (supporting); Writing – review & editing (equal). **Meike Bartels:** Data curation (equal); Investigation (supporting); Project administration (equal); Resources (equal); Writing – review & editing (equal). **Jouke Jan Hottenga:** Conceptualization (supporting); Data curation (supporting); Formal analysis (supporting); Investigation (supporting); Resources (supporting); Software (supporting); Visualization (supporting); Writing – review & editing (equal). **Mariëlle W Pijnenburg:** Funding acquisition (supporting); Supervision (supporting); Writing – review & editing (equal). **Gerard H Koppelman:** Funding acquisition (supporting); Supervision (supporting); Writing – review & editing (equal). **Anke‐Hilse Maitland ‐ van der Zee:** Conceptualization (equal); Funding acquisition (equal); Methodology (equal); Supervision (supporting); Writing – review & editing (equal). **Conor Dolan:** Formal analysis (lead); Investigation (equal); Methodology (equal); Software (lead); Writing – original draft (supporting); Writing – review & editing (lead). **Dorret Boomsma:** Conceptualization (equal); Funding acquisition (equal); Investigation (equal); Methodology (lead); Supervision (lead); Writing – original draft (supporting); Writing – review & editing (lead).

### PEER REVIEW

The peer review history for this article is available at https://publons.com/publon/10.1111/pai.13762.

## Supporting information

Supplementary Material
