# Supplementary material for: Persistence of parental‐reported asthma at early ages: A longitudinal twin study
Source: Pediatr Allergy Immunol. 2022 Mar 12;33(3):e13762. doi: 10.1111/pai.13762 (PMC9314674; doi:10.1111/pai.13762)
Supplement: Supplementary file 1 — Supplementary Material [file PAI-33-0-s001.docx]

Persistence of parental-reported asthma at early ages: a longitudinal twin study

Elise M.A. Slob, PharmD, PhD^1,2,3^

Cristina Longo, PhD^1^

Susanne J.H. Vijverberg, PhD^1,2^

Toos C.E.M. van Beijsterveldt, PhD^4^

Meike Bartels, PhD^4^

Jouke Jan Hottenga, PhD^4^

Mariëlle W. Pijnenburg, MD, PhD^5^

Gerard H. Koppelman, MD, PhD^6,7^

Anke H. Maitland-van der Zee, PharmD, PhD^1,2*^

Conor V. Dolan, PhD^4^

Dorret I. Boomsma, PhD^4^

Supplementary information

Supplementary information 1. Brief description of the quality control and studies used to calculate the asthma polygenic risk score

Quality control

Prior to imputation, quality control was performed on each separate platform (Hardy Weinberg Equilibrium p-value <1×10^-6^, Minor Allele Frequency cutt-off <1×10^-6^, call rate per SNP <0.95 and call rate per individual ≥ 0.90). DNA samples not matching gender, familial Identity By Descent, CEU ancestry or whom had an excess of homo- and heterozygosity (-0.10 < PlinkF > 0.10) were removed. For each platform, data were then converted to build38, aligned to TOPMED imputation panel, phased (Eagle) and imputed (Minimac4) on the Michigan imputation server using internal imputation and data QC protocols^9^. No significant differences were found between APRS scores across the genotyping platforms (p=0.142).

The expected twin correlations of the APRSs are 1 in MZ twins (as they are genetically identical) and 0.5 in DZ twins. APRS correlation equaled one in MZ twins, as expected, and 0.524 in DZ twins, which is close to its expected value of 0.5.

Selected studies

Data of the platforms were merged to single best guess Plink genotypes, and the 26 SNPs were selected. MZ missing APRS were imputed if one MZ twin did have the score and the other did not. A brief description about the included studies from which the 26 SNPs were derived is given below:

Ferreira 2019 Am J Hum^1^

A GWAS in UK Biobank (n=447,628) to identify subtype specific genetic associations of childhood-onset and adult-onset asthma.

Pividori 2019 Lancet Res Med^2^

A GWAS in 376,358 individuals of UK biobank to identify shared and non-shared genetic risk loci of childhood-onset and adult-onset asthma.

Savenije JACI 2014^3^

A candidate-gene study in PIAMA (n=2007) and ALSPAC (n=7247) in which wheezing phenotypes and asthma diagnosis at age 8 were chosen as the outcome. Candidate-genes were from pathophysiological or immunological pathways of IL-33, IL1RL1, IL1rAcP, TIRAP, IL-1 en TRAF6.

Moffatt 2010 NEJM^4^

A GWAS (n=10,365 adults with asthma and 16,110 adults without asthma) in which they stratified for childhood-onset asthma.

Himes 2009 Am J Hum^5^

A GWAS in CAMP (n=359 children with asthma and 846 children without asthma) in which genes were identified related to childhood-onset asthma.

Torgerson Nat Genet 2011^6^

A meta-analysis of GWAS in 5,416 asthma cases representing European Americans, African Americans/African Caribbeans and Latinos, and replicated five regions among the most significant signals in 12,649 individuals from the same ethnic groups.

Moffatt 2007 Nature^7^

A GWAS (n=994 patients with childhood-onset asthma and 1,243 non-asthmatics) to identify associations with childhood-onset asthma and a replication in 2,320 German children and 2,320 British children.

Granell JACI 2013^8^

A candidate-gene study in ALSPAC (n=7045) in which was studied whether earlier associations with childhood asthma were related to specific childhood asthma phenotypes with a focus on the 17q21 region close to *ORMDL3.*

Supplementary information 2. Brief description of the analysis investigating sex moderation at age 3 and 7

We investigated sex moderation by testing whether the twin correlations (r) were different as a function of sex (rMZF = rMZM; rDZM = rDZF = rDZOS). The correlations did not differ, so we infer that there is no sex moderation at age 3 and age 7. To address this question, we fitted the 6 group twin model to estimate the tertrachoric correlations separate in the following groups:

group correlation thresholds

MZM rMZM tM

MZF rMZF tF

DZM rDZM tM

DZF rDZF tF

DZMF rDZMF = rDZFM tM, tF

DZFM rDZFM = rDZMF tF, tM

In the full model we estimate 5 tetrachoric correlations, and two thresholds. Note that we analysed the two phenotypic separately (asthma at age 3 and asthma at age 7).

We tests the hypotheses:

H_1: rDZM=rDZF=rDZMF=rDZFM (a two DF test) (no sex moderation of DZ phenotypic correlations)
H_2: rMZM=rMZF (a one DF test) (no sex moderation of MZ phenotypic correlations)

H_3: rDZM=rDZF=rDZMF=rDZFM and rMZM=rMZF (a 3 DF test) (no sex moderation)

We test each three hypothesis at age 3 with an alpha of .05/3 = 0.0167

We test each three hypothesis at age 7 with an alpha of .05/3 = 0.0167

(This is a compromise: .05 per test is too liberal, but .05/6 per test is too strict)

Results Age 3

Tetrachoric correlations in the 6 groups, no constraints, i.e., 5 correlations (MZM, MZF, DZF, DZF, DZFM=DZMF)

group correlation

MZM 0.952034

MZF 0.9646008

DZM 0.6014834

DZF 0.5690056

DZMF=DZFM 0.5653507

Likelihood ratio tests (LRTs):

H_1: rDZM=rDZF=rDZMF=rDZFM (a two DF test) (no sex moderation of DZ phenotypic correlations)
LRT=0.566 (p=.753; alpha=.0167)

H_2: rMZM=rMZF (a one DF test) (no sex moderation of MZ phenotypic correlations)

LRT=1.336 (p=.247; alpha=.0167)

H_3: rDZM=rDZF=rDZMF=rDZFM and rMZM=rMZF (a 3 DF test) (no sex moderation)

LRT=1.910 (p=.5912; alpha=.0167)

Conclusion: no sex moderation.

Correlations in the model without sex moderation:

group correlation

MZM=MZF 0.9589639

DZM=DZF=DZMF=DZFM 0.5742706

Results Age 7

Tetrachoric correlations in the 6 groups, no constraints, i.e., 5 correlations (MZM, MZF, DZF, DZF, DZFM=DZMF)

group correlation

MZM 0.9063577

MZF 0.9094317

DZM 0.527179

DZF 0.3973618

DZMF=DZFM 0.3474596

Likelihood ratio tests (LRTs):

H_1: rDZM=rDZF=rDZMF=rDZFM (a two DF test) (no sex moderation of DZ phenotypic correlations)
LRT=7.209 (p=.0271; alpha=.0167)

H_2: rMZM=rMZF (a one DF test) (no sex moderation of MZ phenotypic correlations)

LRT=0.019 (p=.888; alpha=.0167)

H_3: rDZM=rDZF=rDZMF=rDZFM and rMZM=rMZF (a 3 DF test) (no sex moderation)

LRT=7.231 (p=.0.648; alpha=.0167)

Conclusion: no sex moderation.

Correlations in the model without sex moderation:

group correlation

MZM=MZF 0.9080032

DZM=DZF=DZMF=DZFM 0.4027951

Supplementary table 1. Selection of single nucleotide polymorphisms based on previous genetic studies

| SNP | position (Build38) | risk allele | reference allele | HWE  p-value | Allele frequency ref. allele | Info | Gene | Study |
| --- | --- | --- | --- | --- | --- | --- | --- | --- |
| rs67551275 | 1:9296617 | C | T | 0.27 | 0.55 | 0.94 | *SPS1* | Ferreira et al. 2019 Am J Hum Genet^1^ |
| rs61816761 | 1:152313385 | A | G | 0.32 | 0.98 | 0.81 | *FLG, HRNR, FLG2* | Pividori et al. Lancet Resp Med 2019^2^ |
| rs7518129 | 1:173194429 | G | A | 0.99 | 0.68 | 0.99 | *TNFSF4, TNSF18, PRDX6* | Pividori et al. Lancet Resp Med 2019^2^ |
| rs10208293 | 2:102349850 | A | G | 0.63 | 0.73 | 1.00 | *IL1RL1* | Savenije et al. JACI 2014^3^ |
| rs13424006 | 2:102350776 | C | T | 0.75 | 0.63 | 1.00 | *IL1RL1* | Savenije et al. JACI 2014^3^ |
| rs10204137 | 2:102351752 | G | A | 0.74 | 0.63 | 1.00 | *IL1RL1* | Savenije et al. JACI 2014^3^ |
| rs3771166 | 2:102369762 | A | G | 0.95 | 0.63 | 1.00 | *IL18R1* | Moffatt et al. NEJM 2010^4^ |
| rs10187276 | 2:227805721 | A,C | T | 0.59 | 0.27 | 0.80 | *SLC19A3, CCL20* | Ferreira et al. 2019 Am J Hum Genet^1^ |
| rs12634152 | 3:188403231 | T | C | 0.28 | 0.51 | 0.96 | *LPP, FLJ42393, LPPAS1* | Pividori et al. Lancet Resp Med 2019^2^ |
| rs1588265 | 5:60073967 | G | A | 0.39 | 0.69 | 1.00 | *PDE4D* | Himes et al. Am J Hum Genet 2009^5^ |
| rs1544791 | 5:60143255 | A | G | 0.41 | 0.69 | 0.99 | *PDE4D* | Himes et al. Am J Hum Genet 2009^5^ |
| rs1837253 | 5:111066174 | C | T | 0.37 | 0.28 | 0.99 | *TLSP/GSDMA* | Torgerson et al. Nat Genet 2011^6^ |
| rs1295686 | 5:132660151 | C | T | 0.00 | 0.21 | 0.98 | *IL13* | Moffatt et al. NEJM 2010^7^ |
| rs2051809 | 5:132721182 | A | C | 0.23 | 0.73 | 0.97 | *KIF3A, IL4, CCNI2* | Pividori et al. Lancet Resp Med 2019^2^ |
| rs274943 | 9:23585841 | C | T | 0.62 | 0.54 | 0.98 | *DMRTA1, ELAVL2* | Ferreira et al. 2019 Am J Hum Genet^1^ |
| rs1342326 | 9:6190076 | C | A | 0.20 | 0.84 | 0.99 | *IL33* | Moffatt et al. NEJM 2010^4^ |
| rs12365699 | 11:118872577 | G | A | 0.03 | 0.18 | 1.00 | *CXCR5, DDX6* | Pividori et al. Lancet Resp Med 2019^2^ |
| rs1887704 | 13:99322238 | G | C | 0.81 | 0.31 | 0.98 | *UBAC2* | Ferreira et al. 2019 Am J Hum Genet^1^ |
| rs3785356 | 16:27337847 | T | C | 0.00 | 0.70 | 0.97 | *IL4R* | Ferreira et al. 2019 Am J Hum Genet^1^ |
| rs9303277 | 17:39820216 | C | T | 0.24 | 0.51 | 1.00 | *IKZF9* | Granell R et al. JACI 2013^8^ |
| rs11557467 | 17:39872381 | T | G | 0.35 | 0.49 | 0.99 | *ZPBP2* | Granell R et al. JACI 2013^8^ |
| rs4795399 | 17:39905186 | T | C | 0.34 | 0.45 | 0.99 | *GSDMB, ZPBP2, ORMDL3* | Pividori et al. Lancet Resp Med 2019^2^ |
| rs2305480 | 17:39905943 | A | G | 0.35 | 0.54 | 0.99 | *GSDML* | Granell R et al. JACI 2013^8^ |
| rs3902025 | 17:39963001 | A, C, T | G | 0.37 | 0.42 | 0.99 | *GSDM1* | Granell R et al. JACI 2013^8^ |
| rs3894194 | 17:39965740 | C | T | 0.81 | 0.46 | 0.98 | *ORMDL3* | Moffatt et al. Nature 2007^7^ |
| rs2284033 | 22:37137994 | G | A | 0.24 | 0.44 | 0.99 | *IL2RB* | Moffatt et al. NEJM 2010^7^ |

HWE: Hardy Weinberg Equilibrium; info: imputation quality of the SNP

Supplementary table 2. Demographics of individual children at age 3 and 7

|  | asthma-like symptoms at age 3  (n=3,087) | no asthma-like symptoms at age 3  (n=27,939) | asthma at age 7  (n=2,273) | no asthma at age 7  (n=22,890) |
| --- | --- | --- | --- | --- |
| Zygosity  MZ  DZ same-sex  DZ opposite-sex  missing | 1,053 (34.1%)  1,027 (33.3%)  1,006 (32.6%)  1 (0.0%) | 9,238 (33.1%)  9,408 (33.7%)  9,260 (33.1%)  33 (0.1%) | 774 (34.1%)  758 (33.3%)  741 (32.6%)  0 (0.0%) | 8,244 (36.0%)  7,396 (32.3%)  7,238 (31.6%)  12 (0.1%) |
| Gender  Male | 1,834 (59.4%) | 13,643 (48.8%) | 1,329 (58.5%) | 11,197 (48.9%) |
| Breastfeeding  None  < 2 wk  2-6 wk  6 wk – 3 mnths  3-6 mnths  > 6 mnths  missing | 1236 (40.0%)  287 (9.3%)  374 (12.1%)  325 (10.5%)  240 (7.8%)  219 (7.1%)  406 (13.2%) | 9,333 (33.4%)  2,464 (8.8%)  4,004 (14.3%)  3,289 (11.8%)  2,562 (9.2%)  2,614 (9.4%)  3,673 (13.1%) | 1,024 (45.1%)  182 (8.0%)  232 (10.2%)  232 (10.2%)  167 (7.3%)  141 (6.2%)  295 (13.0%) | 8,671 (37.9%)  1,859 (8.1%)  2,908 (12.7%)  2,573 (11.2%)  1,944 (8.5%)  1,840 (8.0%)  3,095 (13.5%) |
| Outside home child care  none  1-4h week^-1^  5-8h week^-1^  9-16h week^-1^  17-24h week^-1^  >24h week^-1^  Missing | 219 (7.1%)  359 (11.6%)  713 (23.1%)  612 (19.8%)  479 (15.5%)  183 (5.9%)  522 (16.9%) | 1,961 (7.0%)  2,673 (9.6%)  5,935 (21.2%)  5,230 (18.7%)  4,258 (15.2%)  1,621 (5.8%)  6,261 (22.4%) | 208 (9.2%)  234 (10.3%)  553 (24.3%)  309 (13.6%)  259 (11.4%)  112 (4.9%)  598 (26.3%) | 2,130 (9.3%)  2,058 (9.0%)  5,485 (24.0%)  3,238 (14.1%)  2,607 (11.4%)  1,065 (4.7%)  6307 (27.6%) |
| Educational attainment mother  ≤ 9 years  10-12 years  < 2 years tertiary  ≥ 2 years tertiary  missing | 210 (6.8%)  820 (26.6%)  1,366 (44.3%)  681 (22.1%)  10 (0.3%) | 951 (3.4%)  6700 (24.0%)  11,906 (42.6%)  8,313 (29.8%)  69 (0.2%) | 166 (7.3%)  715 (31.5%)  877 (38.6%)  507 (22.3%)  8 (0.4%) | 1,140 (5.0%)  6,486 (28.2%)  9,214 (40.3%)  5,986 (26.2%)  64 (0.3%) |
| Educational attainment father  ≤ 9 years  10-12 years  < 2 years tertiary  ≥ 2 years tertiary  missing | 224 (7.3%)  929 (30.1%)  1,060 (34.3%)  784 (25.4%)  90 (2.9%) | 1,426 (5.1%)  6,871 (24.6%)  9,936 (35.6%)  9,141 (32.7%)  565 (2.0%) | 199 (8.8%)  689 (30.3%)  723 (31.8%)  611 (26.9%)  51 (2.2%) | 1,374 (6.0%)  6,091 (26.6%) 7,714 (33.7%) 7,277 (33.7%) 434 (1.9%) |
| Atopy  missing | 777 (25.2%)  1,038 (33.6%) | 3,358 (12.0%)  8,374 (30.0%) | 737 (32.4%)  583 (25.6%) | 3,003 (13.1%)  4,756 (20.8%) |
| Older siblings  None  missing | 1,167 (37.8%)  856 (27.7%) | 11,044 (39.5%)  7,873 (28.2%) | 1,026 (45.1%)  417 (18.3%) | 9,917 (43.3%)  3,886 (17.0%) |

Supplementary table 3. Tetrachoric correlations and estimates for asthma at age 3 and 7, estimates for the model with asthma polygenic risk score

|  | MZ (n) | MZ cor | DZ same-sex (n) | DZ opposite-sex (n) | DZ cor | proportion due to A  heritability | proportion due to C | proportion due to E | explained variance (APRS) (%) |
| --- | --- | --- | --- | --- | --- | --- | --- | --- | --- |
|  | | | | | | standardised liability variance components | | | |
| age 3 | 7,329 | 0.954 | 7,177 | 7,035 | 0.555 | 0.797 | 0.156 | 0.046 | 0 |
| age 7 | 7,329 | 0.904 | 7,177 | 7,035 | 0.452 | 0.903 | 0* | 0.096 | 0 |
|  | | | | | | liability covariance | | | |
| age 3 and 7 | r_liability_ (including APRS): 0.746 | | | | | R(A) = 0.806 | NA | R(E) = 0.934 | NA |
| contribution to r_liability_ |  | | | | | 0.684 | NA | 0.062 | NA |
| proportions |  | | | | | 0.916 | NA | 0.083 | NA |

APRS: asthma risk score, MZ: monozygotic, DZ: dizygotic, n: number of twin pairs including twins with missing data (6,612 children with SNP data), cor: correlation, C: shared environment component, E: non-shared environment component, NA: not applicable, R(E): environmental correlation, R(A): genetic correlation. *Fixed to zero. Adjusted for gender.

Supplementary references

1. Ferreira MAR, Mathur R, Vonk JM, et al. Genetic Architectures of Childhood- and Adult-Onset Asthma Are Partly Distinct. *Am J Hum Genet.* 2019;104(4):665-684.

2. Pividori M, Schoettler N, Nicolae DL, Ober C, Im HK. Shared and distinct genetic risk factors for childhood-onset and adult-onset asthma: genome-wide and transcriptome-wide studies. *Lancet Respir Med.* 2019;7(6):509-522.

3. Savenije OE, Mahachie John JM, Granell R, et al. Association of IL33-IL-1 receptor-like 1 (IL1RL1) pathway polymorphisms with wheezing phenotypes and asthma in childhood. *J Allergy Clin Immunol.* 2014;134(1):170-177.

4. Moffatt MF, Gut IG, Demenais F, et al. A large-scale, consortium-based genomewide association study of asthma. *N Engl J Med.* 2010;363(13):1211-1221.

5. Himes BE, Hunninghake GM, Baurley JW, et al. Genome-wide association analysis identifies PDE4D as an asthma-susceptibility gene. *Am J Hum Genet.* 2009;84(5):581-593.

6. Torgerson DG, Ampleford EJ, Chiu GY, et al. Meta-analysis of genome-wide association studies of asthma in ethnically diverse North American populations. *Nat Genet.* 2011;43(9):887-892.

7. Moffatt MF, Kabesch M, Liang L, et al. Genetic variants regulating ORMDL3 expression contribute to the risk of childhood asthma. *Nature.* 2007;448(7152):470-473.

8. Granell R, Henderson AJ, Timpson N, et al. Examination of the relationship between variation at 17q21 and childhood wheeze phenotypes. *J Allergy Clin Immunol.* 2013;131(3):685-694.

9. Das S, Forer L, Schönherr S, et al. Next-generation genotype imputation service and methods. *Nat Genet.* 2016;48(10):1284-1287.
